# Professionals’ experiences with paediatric colonoscopy: an interview study

**DOI:** 10.1186/s12912-019-0331-5

**Published:** 2019-03-11

**Authors:** Vedrana Vejzovic

**Affiliations:** Department of Care Science, Malmö University, Faculty of Health and Society, SE-205 06 Malmö, Sweden

**Keywords:** Nurse, Experience, Paediatric colonoscopy

## Abstract

**Background:**

Colonoscopy plays a crucial role in the diagnosis of paediatric inflammatory bowel disease (IBD), adolescents comprise 25% of all cases of IBD. Several studies have found that a safe, informative, and effective colonoscopy, performed in a child-friendly atmosphere with minimal distress to the child, is difficult to achieve.

The aim of this study was to describe nurse’s experiences of the pre-colonoscopy procedure prior in children.

**Methods:**

A qualitative design with a thematic content analysis approach was used. Fifteen nurses at a children’s hospital participated in interviews regarding their experiences of the bowel cleansing procedure with PEG in children.

**Results:**

Four key themes were extracted from the nurses’ experiences; lack of knowledge, challenges surrounding information, responsibility without control and assembly line- like procedure..

**Conclusions:**

This study shows that nurses feel that they need more time and education opportunities before involved in paediatric colonoscopies.

## Background

Colonoscopy is a routine endoscopic non-surgical investigation of the colon and the outermost part of the small intestine. It is considered effective and safe for children of all ages, including premature new-borns [1, 2]. Colonoscopy is crucial for the diagnosis and monitoring of, paediatric inflammatory bowel disease (IBD) (e.g., [3, 4]). Previous studies have shown that adolescent comprise 25% of all cases of IBD. The prevalence is greater in adolescents between the ages of 15 and 19 years, and the median age of adolescent IBD patients is 15 years [5]. Children with IBD must often undergo a series of diagnostic tests, including abdominal computed tomography, upper endoscopy, and colonoscopy with biopsies [6]. Colonoscopy is normally performed while the child is under anaesthesia [7, 8], which has been found to facilitate the procedure in children [9, 10]. However, the procedure, especially bowel cleansing prior to colonoscopy, can be challenging for the child and accompanying parents. [10, 11]. The nurse’s role is to organize care and prepare the child prior to colonoscopy.

The most important aspect of pre- colonoscopy preparation is bowel cleansing. A variety of bowel cleansing regimens have been evaluated, but for children, the most commonly used preparation is polyethylene glycol with electrolytes (PEG), which is generally recommended by the European Society for Paediatric Gastroenterology, Hepatology and Nutrition (ESPGHAN) working group due to its minimal side effects and good cleansing quality [13]. The recommended intake of PEG is 25–35 ml/kg bodyweight per hour orally, until clear intestinal fluid is obtained [14]. This dosage can require the child to consume between two and four litres of PEG during bowel cleansing.

A safe, informative and effective colonoscopy performed in a child-friendly atmosphere with minimal distress to the child, is the recommended practice in paediatric care [1, 15]. However, several studies have found that this goal is difficult to achieve, largely because of the large volumes of bad-tasting laxative, which both children and parents experience as the most difficult to achive (e.g., [10, 11, 12, 16, 17, 18]). The laxative has been described as tasting ‘disgusting’, ‘bad’, ‘awful’, ‘salty’ and/or ‘like oil,’ and some children have reported that they ‘cheated’ with the laxative [10, 12]. The difficulties with laxative intake may result in an unclean intestine, which can lead to a repeat procedure or failure to detect intestinal changes [3, 4]. Parents are often at the child’s side to provide support during the procedure [11, 12]. Previous studies show that parents often feel responsible for their child’s physical care and emotional welfare when their child is at hospital and are willing to provide basic paediatric care when their child is sick [18–23]. However, the parents do not feel comfortable taking responsibility for bowel cleansing prior to colonoscopy [11, 12] because of the discomfort that bowel cleansing causes their children [11, 19, 20]. When parents were interviewed regarding their child’s pre-colonoscopy preparation, they stated that they were forced to actively participate in the procedure without specific training and that they felt uncomfortable in this situation [11]. Previous studies [10, 11] have shown that bowel cleansing with PEG in children can present unique challenges for both the children and their parents because of the procedure’s complexity. Optimizing the pre- colonoscopy procedure for children requires collaboration between the child, parents and nurses [10, 11]. The results of these studies show that both the children and their parents lack nursing guidance during the pre- colonoscopy procedure. Because of the need for collaborate among the children, nurses and parents, it is also important to understand nurses’ experiences of the procedure. Therefore, this study was aimed describe the nurses’ experiences of the pre- colonoscopy procedure in children.

## Methods

This qualitative study using interviews with nurses was conducted at the Paediatrics Department, Skåne University Hospital, Malmö Sweden.

### Participants and setting

The participants consisted of 15 nurses with experience in pre- colonoscopy preparation in children. Purposive sampling, that considered age, years in the nursing profession and years in paediatric care was applied to ensure the possibility of collecting a broad range of their experiences. The nurses involved in children’s colonoscopies had different professional backgrounds. Registered nurses RNs who hold a bachelor’s degree in nursing science, nurse specialists RSCNs who have completed a nurse specialist programme in paediatric care, and assistant nurses AN (certified nursing assistant), all referred to as nurses henceforth, are usually involved in all aspects of preparing children for colonoscopy. At the time of the study, approximately 100 children (0–18 years of age) per year were prepared for colonoscopy at the Paediatric Department at Skåne University Hospital. Bowel cleansing is performed using PEG, and the entire preparation procedure takes place over the course of two days with the child as an inpatient. The procedure begins with a diet regimen at home.

### Data collection

A written agreement to conduct the study was provided by the Paediatric Department at the University Hospital in southern Sweden. Written information in the form of a poster was posted at the clinic, and nurses involved in pre- colonoscopy preparation were asked to participate in the study. The interviews were conducted from September 2016 to March 2017 by the author. The participants were allowed to choose the time and the preferred setting for the interview during their working hours. Each interview began with providing oral information to the participants and asking for their informed consent. Individual semi-structured interviews were conducted in a secluded parlour at the hospital. The questions were groupt into specific areas: overall experiences of preparing children for colonoscopy, experiences of bowel cleansing with PEG, experiences of information and experiences of the participating parents. These topic areas were based on results from earlier studies that took the perspectives of the children and parents [10, 11]. All the interviews started with an opening question to collect demographic data, and proceeded with an open question that prompted the participants to describe “experiences when they prepared a child prior to an elective colonoscopy,” “the procedure’s complexity” and “how to optimize the procedure prior to colonoscopy for children”.

### Data analysis

Data analysis was conducted using thematic content analysis as described by Braun and Clarke [26]. The author, an academic researcher who was not involved in clinical work, performed all the interviews, coded each segment of the text and organized the content into preliminary themes. To maximize reliability, reduce bias and, ensure that the coding was structured and consistent with the research question the initial coding framework was refined and reviewed by two RSCNs. The grouping of codes into themes occurred through team discussion. This stage also involved reflections on whether the themes were convincing, and examinations of the relationships between the themes. The reflection process was repeated until the essence of the themes was defined. The result are presented as an analytical story with representative quotes to present the essence of nurses’ experience of paediatric colonoscopy and the preceding procedures.

### Ethical considerations

The study was approved by the Regional Ethical Review Board in Lund (Ref. No. 2012/ 186). The participants were informed, in accordance with the Declaration of Helsinki (World Medical Association, 2013), that their participation was voluntary and that they could withdraw their participation at any time without any negative consequences. The participants were also informed both orally and in writing about the aim of the study before they gave their consent to participate.

## Results

### Participant and context characteristics

Data were obtained from six RNs, three RSCNs and six ANs. All the participants were women, and they had a mean age of 40.7 years (range 24–64 years). The work experience of the participants ranged from seven months to 41 years (mean = 17.2), with a mean of 5.2 years for the RNs, 17.5 years for the RSCNs and 28.6 years for the ANs. Bowel cleansing prior to colonoscopy was performed at a paediatrics department where children with various medical and surgical diagnoses are inpatients. Colonoscopies were performed at the endoscopy department.

### Key themes

The data were organized into four key themes: Lack of knowledge, challenges surrounding information, responsibility without control and an assembly line- like procedure. The nurses´ experiences of preparing children for colonoscopy described in the current text and illuminated by selected quotes from the interviewees.

### Lack of knowledge

The nurses felt that they routinely performed pre- colonoscopy bowel cleansing in children based on written manuals. They experienced a lack of knowledge regarding the illness (IBD) and its symptoms; however, the majority of those interviewed stated that they had a good understanding of the pre- colonoscopy procedure. The nurses experience reported that they had knowledge of the procedure (e.g., bowel cleansing), but they lacked knowledge and training about paediatric colonoscopy. Irrespective of the knowledge that these children have gastrointestinal symptoms, they are often perceived by nurses as alert and unaffected by illness when they come to hospital to undergo a colonoscopy. The nurses education is based on the experiences of others.

“*I have prepared many children for colonoscopy but don’t truly have any specialist training for it. We help each other and of course we use manuals…Now I’m wondering whether that’s enough…I would like to have more medical knowledge of the diseases that form the basis of the examination…*” (IP 3).

The majority of the nurses lacked extensive practice, given that they are required to prepare children of different ages with varying levels of maturity and experiences. Some of the informants had participated in a few colonoscopy examinations and felt that this experience gave them a better understanding of the importance of careful preparation for the examination. The nurses also found that they worked with different complex procedures often simultaneously, and that they always followed the written instructions but often did not have the time to reflect on them. *“We know that this has to be done and we do it in accordance with the rules… but I don’t often think about why it has to be done this way…I wish we had more time for training on the different procedures we perform on children”* (IP1). They stated that they lacked the necessary training and specific practices when they performed the procedure the first time, and they relied on each other’s experiences and written templates.

### Challenges surrounding information

Thoughts regarding information and its scope were repeated several times. Parents are often perceived by nurses as well informed and positive about the examination; however, they are often sad and worried about what the child will experience. The nurse’s experiences regarding information were varied. Some stated that they often thought about whether the information provided regarding the preparations was sufficient and covered all the aspects, as the children do not always seem to know what will happen and why when they come to the hospital. Some of the informants felt that the majority of children are well prepared and know that they need to drink a large amount of fluid. However, it was found that doing so was not often as easy as the children thought and was difficult to handle. Information about the colonoscopy examination is provided in advance, both in writing and verbally, but the nurses indicated that often, needs to repeated in full from the start, and adequate time for this repetition is not been appropriately allocated.

*“I often feel that I need to inform about everything at the start….they often ask question as if no information has been provided and …well…I know that the doctor has provided it and in writing…I know myself that they’re not always receptive and that the questions will come later…it always goes better when they participate and know about what will happen…”* (IP 11).

The participants indicated that they know that the child and parents receive both written and verbal information regarding the procedure; however, they felt that the child does not always understand the information. Such children find it more difficult to handle the preparations and may be a “*tough nut to crack*” (IP 12). The participants also reported that parents have “*often done their homework*” (IP 8), and that it is always easier to get the child to cooperate if the parents are knowledge about the procedure. “*It feels as though many children who come to us are unaware of what will happen. It seems as though some are completely shocked when they learn that they need to drink a lot or when we mention the tube. They keep trying incessantly to consume the Laxabon (PEG) at any cost to avoid the tube. This also means that the procedure takes a long time and in the end we need to use a tube anyway*.” (IP 15).

At the end of their interview, the nurses often reflected the children’s preparation for the procedure, and most of them emphasized that they feel that the procedure was performed at the same way for everyone.

*“It feels like I do something as though everyone is the same person. We have tools that we use, but the children are actually not willing to do it; we impose extremely difficult demands on them...who wants to use laxative, they need to decide whether they want a tube...well, we often forget that it’s difficult to undergo this examination.” (IP 14).*

### Responsibility without control

The nurse’s narratives clearly indicated that bowel cleansing with PEG is perceived as the most difficult part of the procedure for the child and most challenging aspect for itself, as encouraging a child to drink a large amount of bad-tasting fluid is a “major experiment”. The nurses described feeling pressure in terms of ensuring that the intestines were clean, but they did not think that they can control the bowel cleansing.

“*It is expected that the intestines should be clean for the examination and I find it hard to know whether everything is as it should be…there isn’t any way for me to know. I can only assume that the child follows the instructions and ask…nothing else…*” (IP 1).

The participants frequently mentioned that responsibility for the procedure was shared with the parents. “*We share the responsibility with the parents; this is natural*…” (IP 11). They viewed this approach as being natural because of the best interest of the child but also felt that it reduced their ability to control the procedure, which involves a tight timeframe. “*We have a limited amount of time for this procedure, and if the child doesn’t drink the laxative in accordance with the recommendations, there is a greater risk that the examination cannot be performed as planned*” (IP 5).

“*The parents help out and this is for the child’s best interest …the children are less stressed”* (IP 3).

Some of the informants stated that they understood the children’s reluctance to drink the laxative fluid as they have tasted it themselves and found difficult to consume. One of the interviewees said, “*I’ve tried it myself and couldn’t even handle one glass, and I’m supposed to be the mature one*.” (IP 9). The nurses said that in most cases, the children were well behaved and willing to cooperate. Additionally, the nurses indicated feeling that the procedure imposes high demands on the children and their accompanying parents.

“*Well…I must say that many times I feel that the children find it much harder than we anticipate. For us, it’s something that the children need to do, but they are the ones who need to go through it. I often feel that we portray the procedure as being easier than it is. I haven’t heard of anyone who found it easy, but we can’t truly understand how these children truly feel; we take care of those with many different needs*” (IP 2).

Most of the intervieweers felt t.hat the parents were willing to assume an active role in preparations and that they rarely asked for help or imposed any demands. The nurse reported that when the child’s best interest was considered, it was relatively easy to involve parents and believe that the responsibility was shared. The nurse stated that time constraints prevented them from establishing a favourable nurse-patient relationship and that the parent’s presence was considered to be a rescuing factor.

Parents were viewed as assets and rarely as a difficulty. The nurse viewed the parents as assets because of the limited time they could spend with the child.

“*They are, well...I feel that most are eager for the examination to be conducted and they are particularly involved in the drinking process. They truly want the child to be able to cope with the preparations and they are supportive, but I can’t imagine that they truly understand how difficult it must be for the child…they want the child to do the examination, but unfortunately, they don’t always understand how difficult it can be.*..” (IP 6).

The nurses also reported thinking that the responsibility for the child’s preparation was divided between them and the parents. The nurses indicated that rarely parents unwilling to participate in the preparations, and they are expected to ask questions if something is unclear. The nurse stated that before the child starts the procedure, a dialog is conducted with both the child and parent about what will happen. Unless the nurses experience shared responsibility as positive factor, their stressful work situation caused them worry that they did not have proper control of what happened during the procedure.

### An assembly- line like procedure

The nurses identified colonoscopies and their preparation as usual procedures that are performed daily. They stated that the daily stress of their jobs and the fact that the children were rarely very ill when they came to the department for bowel cleanse before a planned colonoscopy did not prompt them to reflect deeply about the procedure. “*It’s like every one is the same person and of course that is not the case /*…/” (IP 14).

Nurses provide daily care for children with different severities of illness and medical diagnoses; most of these children are inpatients, and therefore, their care is prioritized. Thus, the children who need to undergo colonoscopies are perceived by nurses as healthier and are under-prioritized.

“*Unfortunately, sometimes it’s the children who are inpatients and sick who we prioritize, I truly wonder if that’s the right thing to do…after all, these healthy- looking children also need us…yes…*” (IP 6).

The fact that the colonoscopies are usually performed under anaesthesia was considered good for the child. “*The children behave well and the colonoscopy examination can be performed quickly when they are asleep. They don’t tend to have any difficulties afterwards and return home happy. I think ...or...we rarely ask what they truly think about this...Now that I think about it, we should ask about how it was...*” (IP 12).

The informants usually concluded their interviews by saying that the pre- colonoscopy procedure is actually difficult and extensive. They also highlighted that these children often have good health when they come to the hospital and can handle the procedure without any major problems. “*I can feel that we do it like an assembly line and don’t see the individual; we are so accustomed to doing it that the individual is forgotten. Especially regarding teenagers...they look like adults and we expect a lot from them*” (IP 19). Additionally, it was revealed that nurses rarely ask about child/ parents´ experience of the preparation procedure and/or colonoscopy.

## Discussion

The aim of this study was to describe the nurses’ experiences of the pre- colonoscopy procedure in children. Four key themes were extracted from the nurses´ experiences of this procedure and are discussed in relation to the need to facilitate the experiences of the children and their parents prior to colonoscopy.

The present study characterized early recognizable clinical problems that nurses experience in when care of paediatric patients prior to colonoscopy. The results of this study highlight nurses descriptions of their experiences of the pre- colonoscopy procedure in children. To summarize, the nurses’ experiences were based on two factors; their general view of paediatric colonoscopy and their experiences related to the children and their parents during the procedure. Nurses are important healthcare team members involved in planning and providing paediatric care, and thus, it is important to examine their experiences to acquire knowledge and to increase their opportunities to improve care. This assessment is especially important, as we know that neither the children’s nor the parents’ experience of the procedure is positive [10–12] and we need to do something to reduce discomfort for both of them. Previous research shows that professionals and patients do not always have similar thoughts regarding how to provide care during various procedures [27]. The nurses in this study highlighted the difficulties of the pre-colonoscopy procedure; however, these findings were only partially consistent with previous study results from the children’s and parents’ perspective [10–12]. The nurses in this study expressed experiencing limited time and theoretical knowledge of the procedure, and these issues negatively impact the patients’ or caregivers’ experiences. When the present study was planned, the idea was to interview the RSCNs who were involved in this procedure. However, the informants in this study also belonged to other groups of nurses because of variations in practices regarding paediatrics bowel preparations in different contexts. The nurses had access to a description of the procedure that was formulated specifically for the pre- colonoscopy preparation, but they are not required to undergo for education. The fact is that the practices regarding bowel cleansing preparation varies from clinic to clinic, and this variation may be questioned. However, the lack of guidelines within the area and shortages of staff with relevant education are a realities. Comparable results have been obtained in Canada, where findings by Muthiah et al. [28] revealed significant regional differences in the practice of after-hours endoscopy, irrespective of the recommendation by recent Canadian and international guidelines that appropriate staffing for emergency endoscopies essential.

The results of the present study showed that nurses are aware of the difficulties that bowel cleansing with PEG for a planned colonoscopy may entail for the child. Additionally, they considered the entire procedure relatively easy, as the parents help the child and are with the child. The nurses stated that they lacked knowledge regarding the child/parent’s satisfaction with the preparations but felt that everyone was satisfied overall. Based on previous research demonstrating that children do not talk much with nurses during and after the procedure [10], it is worth wondering whether the nurses have a false perception of the child’s satisfaction. The results of previous studies have shown that children generally report negative experiences with nursing care [29], which confirms the existence of a discrepancy between the child’s and the nurse’s experience of care. This study highlighted that a part of the nurse´ pre- colonoscopy work is usually performed routinely, with no time for reflection. Previous research has also shown that both children and their parents feel that care is not individualised [24, 25]. These existing problems cannot be solved without a thorough understanding of the regional problems and political and economic pressures, that can lead to poorer quality of health care. Despite regional recommendations that nurse specialists provider paediatric care, such care duties have to be discharged due to a lack of specialists. This issue may affect the opportunity to take time for reflection as nurses much devote time to time performing several different tasks for wich they are not trained.. The nurses are not satisfied with their work, and they must rely on parents’ to perform parts of the procedure; however they also consider that the parents´ efforts are the child’s best interests, and therefore, they accept the loss of control.

The respondents in this study perceived the parents as a source of support for the child and a resource for the procedure, but previous research has shown that parents feels they were assigned a role with which they were uncomfortable [11]. This problem is widely recognized and has been described in other studies [19; 20]. It is common for nurses and parents not to specifically discuss their roles in the child’s care or care activities during a hospitalization [19, 20]. Previous studies also show that the needs of parents and children, when the child undergoes bowel cleansing, are not always known by nurses [10, 11].

The fact that the interviews were conducted in a department where nurse specialist, registered nurses and assistant nurses work on the same tasks in the field of colonoscopy and the different education backgrounds and experiences of these groups of nurses could be considered limitations of this study because recommendations indicate that only specialist should be responsible for paediatric care. Dury et al. [30] provided evidence of a lack of specialist competence in nursing care in Europe. Their results emphasized the need to improve standards for the education, certification and regulation for specialists in European nursing [30]. However, the lack of specialist nurses is a reality in Sweden, and therefore, it is important to describe the phenomenon based on the prevailing conditions. The results might have been different if only specialists performed this paediatric procedure, but no information gap based on the participants´ educational background could be identified in this study based on the informant’s descriptions.

### Strengths and limitation

The recommendations of Lincoln and Guba [31] regarding credibility were followed. Authors with experience conducting qualitative interviews performed and transcribed all the interviews. The final results were analysed by two respondents who were willing to participate in the analysis, which contributes to greater credibility. Confirmability was verified using quotes from the respondents as presented above. The interviews were conducted in a department where nurse specialists, general nurses and assistant nurses work on the same tasks in the field of colonoscopy and given the different educational backgrounds and experience levels of these groups nurses, we assume that their experiences may be different. However, no such differences could be identified in the present study. The selection of nurses from just one department can be considered as a limitation of this study; however, the participants experiences make them representative of the health care professionals who are involved in caring for children prior to colonoscopy.

## Conclusion

The findings of the present study indicated the different factors that can impact a child’s care prior to colonoscopy. The results indicated that nurses need more knowledge regarding children’s and parents’ needs when a child is in hospital to pre- colonoscopy bowel cleansing, as well as more time to reflect about optimizing each child’s experience. To satisfy children and parents during the procedure, the existing barriers, especially the knowledge gap, must be minimized. The identification of these barriers can lead to effective steps to increase nurses´ knowledge and to create opportunities for better cooperation between children, parents and nurses. The results confirmed the importance of considering all perspectives, those of the parents/caregivers and children as well as the nurses themselves when nurses plan the procedure prior to the paediatric colonoscopy. More research is needed to evaluate practices regarding paediatric colonoscopy with PEG.

## Abbreviations

AN: assistant nurse
IBD: inflammatory bowel disease
IP: interviewed person
PEG: polyethylene glycol
RN: registered nurse
RSCN: nurse specialist
